# Results of venous reflux treatment with 1,470 nm endolaser and correlation with degree of venous insufficiency

**DOI:** 10.1590/1677-5449.200172

**Published:** 2021-04-28

**Authors:** Ana Paula Pires Silva, Daniel Mendes Pinto, Vanessa Aline Miranda Vieira Milagres, Leonardo Ghizoni Bez, Júlio César Arantes Maciel, Caetano de Souza Lopes

**Affiliations:** 1 Hospital Felício Rocho, equipe de Angiologia e Cirurgia Vascular, Belo Horizonte, MG, Brasil.; 2 Faculdade Ciências Médicas de Minas Gerais, Belo Horizonte, MG, Brasil.

**Keywords:** varicose veins, laser therapy, saphenous vein

## Abstract

**Background:**

Patients with advanced chronic venous disease are more likely to need additional procedures for relapsed varicose veins. It has not yet been established whether severity of venous insufficiency is a factor that influences the occlusion rate of saphenous veins treated with endolasers.

**Objectives:**

To analyze occlusion rate of venous segments treated with endolaser and correlate it with patients’ Venous Clinical Severity Score (VCSS) and Clinical-Etiological-Anatomical-Pathological (CEAP) classification.

**Methods:**

Retrospective analysis of a cohort of patients operated using a 1,470 nm endolaser from November 2012 to March 2020. Descriptive statistics were calculated and Kaplan-Meier survival curves were plotted with Cox regression for groups stratified by VCSS and CEAP.

**Results:**

A total of 180 venous segments were analyzed in 170 patients. Mean age was 44.3 ± 9.2 and the majority of patients were female (71%). Mean energy density used in the great saphenous vein was 49.2 ± 8.3 J/cm. The most common complications were pain along the course of the saphenous vein (12.2%) and paresthesias at 6 months (17.2%). There was no difference in venous occlusion rate between groups with VCSS ≤ 7 and VCSS > 7 (p = 0.067). A group of patients classified as CEAP classes C4, C5, or C6 had a lower occlusion rate than a group at classes C2 or C3 (hazard ratio [HR] = 3.22; confidence interval [CI] 1.85, 5.61; p = 0.001].

**Conclusions:**

The occlusion rates of venous segments treated with endolaser were lower in patients with higher CEAP classes. It is probably necessary to use more energy in these patients to achieve effective treatment of saphenous veins.

## INTRODUCTION

Lower limb varicose veins are often secondary to saphenous vein insufficiency. From 37 to 46% of patients with chronic venous disease have insufficiency of the superficial vein system.^1^ Intravenous thermal ablation with lasers (also known as endovenous laser ablation, EVLA) or with radio frequency are minimally invasive techniques that attenuate some of the disadvantages associated with conventional surgery, such as hematomas, scarring, inguinal neovascularization, and extended recovery times during the postoperative period.^1^ The procedure is also less invasive and can be performed in outpatient settings, using local anesthesia only.^2^ EVLA or radio frequency ablation are considered first-line treatments for saphenous vein reflux.^2^^,^ 3

The diameter of saphenous veins is unrelated to the degree of patients’ symptomology,^1^ but studies have shown that great saphenous veins larger than 5.05 mm have a positive predictive value for saphenous vein incompetence and may have a weak relationship with disease stage.^1^^,^ 4 Patients with venous disease characterized by Clinical-Etiological-Anatomical-Pathological (CEAP) classes C3 to C6 exhibited 2 to 3 times greater chance of recanalization over a 1 year period.^5^^,^ 6 However, it has not been determined whether advanced venous disease can interfere in the results of the intravenous ablation technique or even if there is a greater risk of complications in these patients.

The objective of this study was to analyze occlusion rates of saphenous veins treated with a 1,470 nm endolaser and determine whether rates differed depending on degree of venous disease according to Venous Clinical Severity Score (VCSS) and CEAP class. We also analyzed whether there were differences in rates of complications and of saphenous vein recanalization over 7 years of patient follow-up. We defined the hypothesis to be tested as the existence of a correlation between degree of venous insufficiency and occlusion rate of saphenous veins treated with EVLA.

## METHOD

This is a non-concurrent cohort study of patients with varicose veins related to saphenous vein insufficiency who were treated with EVLA. A retrospective analysis was conducted of patients with chronic venous insufficiency treated with the EVLA technique from November 2012 to March 2020. Data were collected from hospital electronic patient records and from the vascular surgery team’s own records. The project was approved by the institutional Ethics Committee under decision number 4.027.149.

The sample size was based on a nonparametric analysis of the sample, with a significance level of 0.05%, statistical test power of 80%, and medium effect size. The result showed that the minimum number of patients in each group to enable comparisons between groups would be 88, making a total sample size of 176.

Patients were included in the study who had undergone EVLA of great or small saphenous veins, accessory veins, and intersaphenous communicating veins. The exclusion criteria were patients aged less than 18 years or more than 80 years and patients with saphenous veins larger than 12 mm.

The surgical technique employed was EVLA, with a 1,470 nm endolaser and a 600 micron radial fiber. Target linear intravenous energy density (LEED) was 40 to 60 J/cm. Patients underwent concomitant phlebectomy after ablation of saphenous veins. All patients underwent tumescence along the path of the veins being treated, with saline mixed or not with xylocaine 2% and no vasoconstrictor and were put in the Trendelenburg position during thermal ablation. Procedures were conducted in a surgical suite during the morning and patients were discharged after the procedure after an observation period of 3 to 6 hours.

Clinical data analyzed were sex, age, venous segments involved, venous insufficiency grade by the CEAP classification, VCSS classification, and presence of venous reflux. Variables on the type of treatment included quantity of laser energy administered, measured in J/cm, the segment treated, and the type of anesthesia administered. Postoperative variables analyzed were presence of venous reflux seen on Doppler ultrasound of the venous segment treated, early and late postoperative complications, and patients’ principal symptoms.

Follow-up examinations were conducted in consulting rooms or an ambulatory clinic by the research team. Follow-up visits were scheduled for 7 days, during the postoperative period, 30 days, and then every 6 months thereafter. At each visit, patients underwent clinical reassessment and Doppler ultrasound examination of the lower limbs. Relapse of reflux in the venous segment treated was defined as presence of reflux lasting more than 0.5 seconds in response to muscle compression maneuvers with the patient standing upright. Relapse of reflux at the level of the great saphenous arch was defined as presence of reflux beyond 5 cm from the saphenofemoral junction.^7^

We calculated descriptive statistics for the data and studied the binary outcome presence or absence of venous reflux in the segment treated with endolaser. Patient data were divided into six groups according to CEAP clinical classification. Kaplan-Meier event-outcome curves were plotted for each CEAP classification. Differences between groups were analyzed with the log-rank test and hazard ratios (HR) were calculated with 95% confidence intervals for each group using Cox multivariate regression. Data on severity of venous disease measured with the VCSS were also divided into groups and analyzed with Cox multivariate regression. The significance level for definition of statistically significant differences was set at 5%. The statistical software programs used were Graphpad Prism version 8 (GraphPad Software, California, United States) and Minitab version 17 (Minitab, LLC, Pennsylvania, United States).

## RESULTS

**Figure 1:**
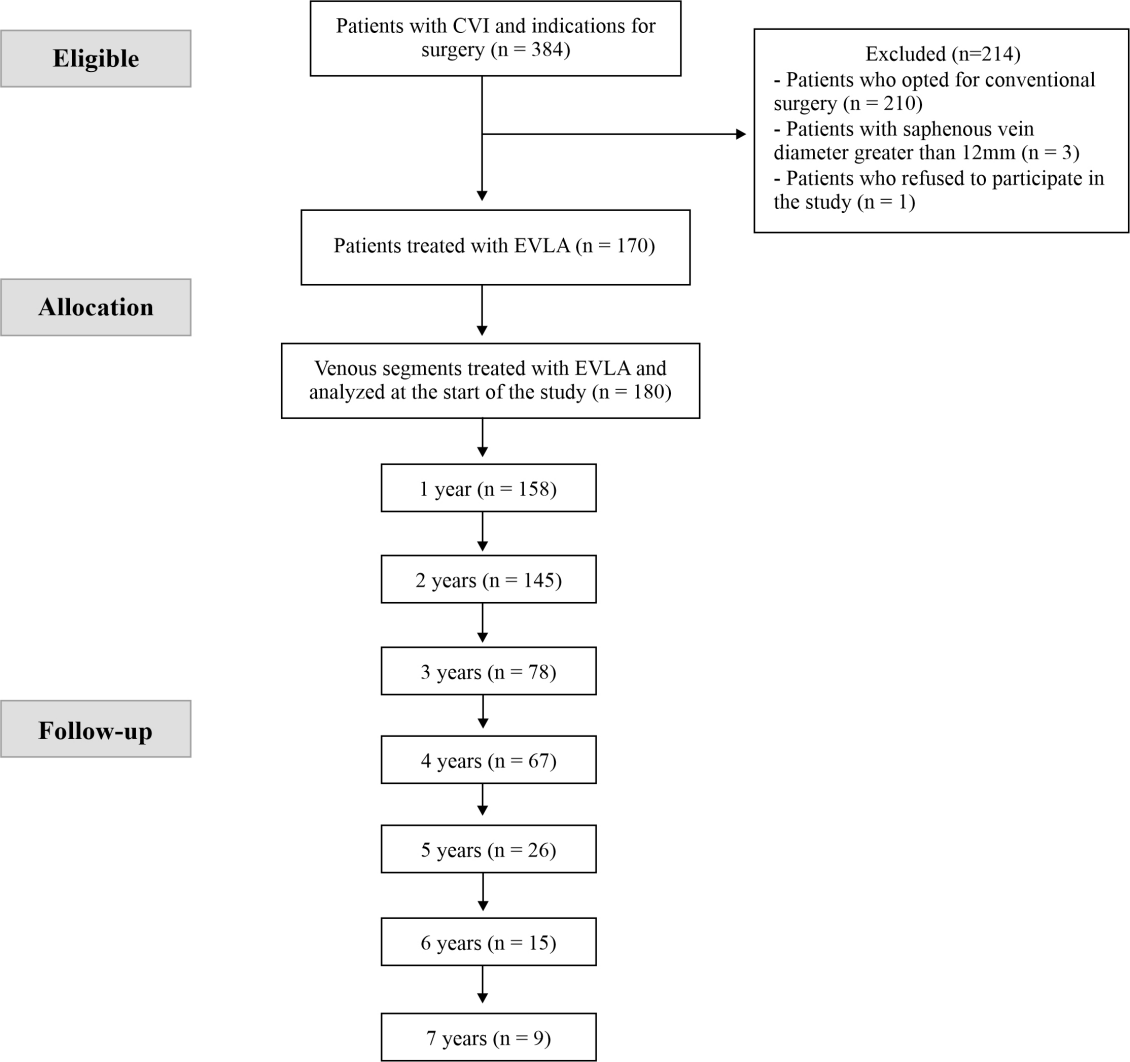
Flow diagram of patients subjected to treatment with endovenous laser thermal ablation (EVLA). CVI = chronic venous insufficiency.

From November 2012 to March 2020, a total of 384 patients were considered eligible. Patients were eligible if they had chronic venous insufficiency and indications for surgical intervention. A total of 180 venous segments were treated with EVLA in 170 of these patients. Figure 1 illustrates the numbers of patients included and excluded and their follow-up durations.

The patient sample included 120 women (71%) with a mean age of 53.68 ± 13.77 years and 50 men (29%) with a mean age of 50.10 ± 13.14 years (p = 0.89). The anesthesia used in 102 cases was local anesthesia with anesthetic sedation (60%); in 40 cases spinal anesthesia was administered (23.5%); and general anesthesia was used in 28 cases (16.5%). Table 1 lists the distribution of venous segments treated and the energy administered. The great saphenous vein was the venous segment most frequently treated and the mean energy applied was 49.12 ± 8.32 J/cm.^2^

**Table 1:** Distribution of the 180 venous segments treated with 1,470 nm endolaser and the energy levels employed.

Venous segment treated	n (%)	Energy density
		(LEED) (J/cm^2^)
Great saphenous vein	161 (89)	49.12 ± 8.32
Small saphenous vein	16 (9)	29.22 ± 5.11
Accessory saphenous vein	2 (1)	27.60 ± 10.55
Intersaphenous communicating vein	1 (1)	25.11 ± 5.23

LEED: linear intravenous energy density (mean ± standard deviation).

The most common complication was paresthesia remaining 6 months after treatment, which occurred in 17.22% of cases, followed by pain along the path of the saphenous vein, in 12.22%. There were three cases of deep venous thrombosis, all diagnosed between 7 and 30 days after surgery (Table 2).

**Table 2:** Complications after thermoablation with 1,470 nm endolaser in 170 patients.

Complication	n (%)
Paresthesia at 6 months	31 (17.22)
Pain along the path of the great saphenous	22 (12.22)
Pigmentation persistent beyond 6 months	12 (6.67)
Deep venous thrombosis	3 (1.67)
Skin necrosis	0
Pulmonary embolism	0

Table 3 lists occlusion rates of the venous segments over the course of follow-up. At 30 days, 175 of the 180 venous segments treated were occluded according to Doppler ultrasound examination, equating to a venous occlusion rate of 97.22%. The occlusion rate of venous segments remained above 95% until the 2-year follow-up. After 2 years, a decrease is observed in the number patients who were followed-up, coinciding with a reduction in the occlusion rate to values below 90%.

**Table 3:** Rate of venous occlusion over the course of follow-up of 180 venous segments treated with 1,470 nm endolaser.

Follow-up	n	Segments occluded	% occlusion
30 days	180	175	97.22%
6 months	178	176	98.88%
1 year	158	155	98.10%
2 years	145	141	97.24%
3 years	78	69	88.46%
4 years	67	60	89.55%
5 years	26	22	84.62%
6 years	15	13	86.67%
7 years	9	8	88.89%

**Figure 2:**
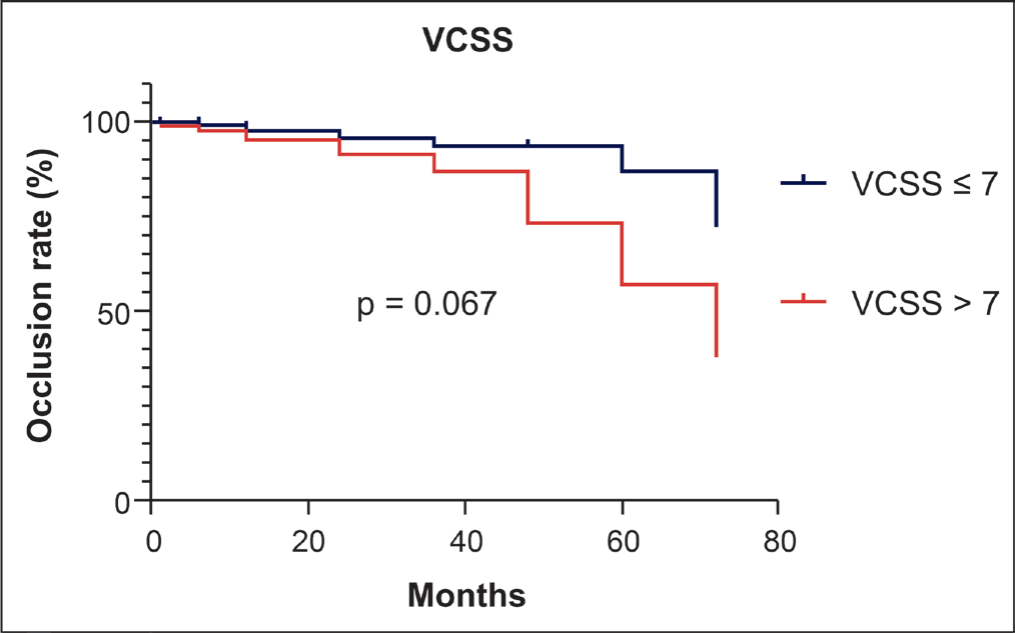
Occlusion rate of venous segments in groups split by median Venous Clinical Severity Score (VCSS).

The median VCSS for the 170 patients was 7. The patients were analyzed in two groups: those with VCSS ≤ 7 and those with VCSS > 7. The occlusion rates of venous segments in the two groups were analyzed. Figure 2 illustrates a temporal analysis using a Kaplan-Meier event-outcome curve, comparing the results in the two groups split by median VCSS. There was no difference in occlusion rates between those with VCSS ≤ 7 and those with higher VCSS over the first 5 years of follow-up (p = 0.067).

**Figure 3:**
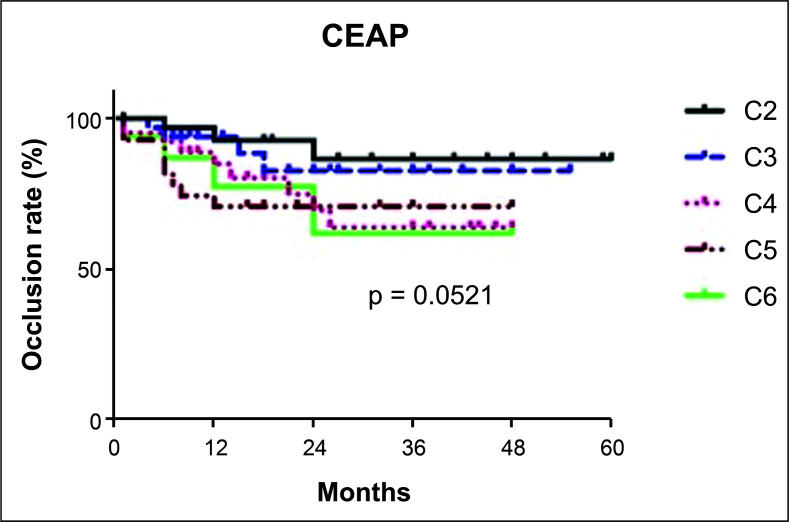
Occlusion rate of venous segments by Clinical-Etiological-Anatomical-Pathological (CEAP) classes.

Figure 3 illustrates the results for occlusion of venous segments by CEAP clinical classification classes. When analyzed individually and over the first 5 years of follow-up, there were no differences between classes C2 to C6 in terms of venous occlusion rates.

**Figure 4:**
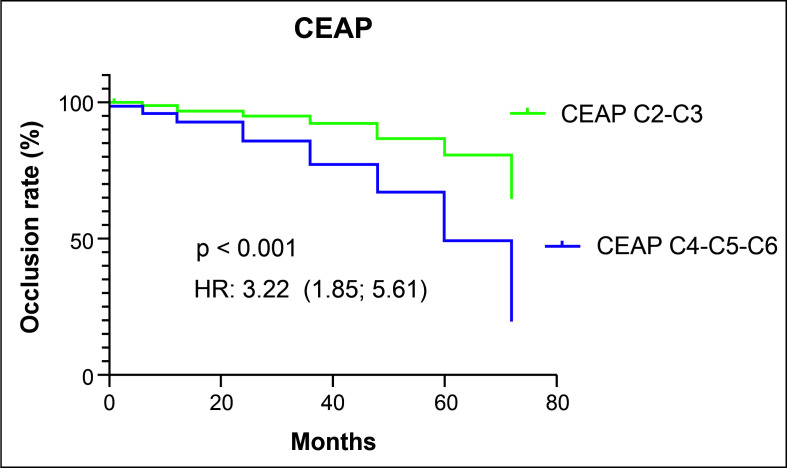
Occlusion rate of venous segments in groups defined by clinical classification. CEAP = Classification Clinical-Etiological-Anatomical-Pathological (CEAP); HR: hazard ratio.

When patients classified at C2 or C3 were analyzed together, they had a higher rate of venous segment occlusion than the group of patients classified at C4, C5, or C6 (p < 0.001; HR = 3.22). During the first 72 months of follow-up, the occlusion rate in the group with C2 or C3 was 3.22 times higher that in the group of patients with C4, C5, or C6, as illustrated in Figure 4.

## DISCUSSION

This study shows that there was an association between more advanced CEAP classification classes and lower rates of occlusion of saphenous veins treated by EVLA. However, the severity of venous insufficiency measured by the VCSS did not have an influence on the result of thermal ablation over time. We also observed that venous segment occlusion rates reduced over the course of follow-up.

Our data show that patients with lipodermatosclerosis, healed ulcers, or active ulcers of the lower limbs had a 3.22 times greater risk of recanalization than patients at the initial venous insufficiency stages. In another study, the authors demonstrate that in patients with venous insufficiency the intimal and medial layers of varicose veins are significantly thicker, with a progressive character as venous disease advances.^5^ Thickening of the vein wall could result in less transmural injury, with subsequent recanalization of the vein after EVLA.^8^ Additionally, patients with C3 to C6 clinical classes develop continuous venous hypertension and are more prone to remodeling of the venous walls. This hypertension leads to histological changes known as hypertensive venous microangiopathy, activating the inflammatory cascade and propagating to the microcirculation and surrounding tissues.^9^ It is possible that patients with advanced venous disease have histological changes that reduce fibrotic transformation after the laser energy is transferred to the venous wall.

The VCSS takes into account clinical symptoms reported by patients, which results in variable information on each patient, such as, for example, the degree of pain and the impact of the disease on routine activities. Studies indicate that patient symptomology and the VCSS classification do not have a directly proportional relationships with disease stage.^1^^,^ 4 These variations could have influenced the results, explaining our finding that an advanced VCSS does not interfere in recanalization over the long term.

The type of anesthesia most frequently used in this patient cohort was local anesthesia with sedation, which is linked to the less invasive procedure, with faster postoperative recovery, and feasibility for use in outpatient settings. All procedures were performed in a hospital, and the cases with general anesthesia were performed using a laryngeal mask and deep sedation. The Vascular Quality Initiative register shows that 54.4% of treatments for varicose veins in the United States were performed in office-based settings with similar resources to hospitals and 37.1% were performed in hospital environments.^10^

LEED has become the reference metric for physicians to calculate the energy delivered. Recommendations in the literature vary from 40 to 90 J/cm of linear energy for the great saphenous vein.^1^ The mean LEED used in our patients, 49.12 ± 8.32 J/cm, was compatible with data from other authors. Flow velocity, venous wall histology, vein caliber, quantity of tributary veins, power, and type of fiber used can all influence the quantity of energy necessary.^1^

The energy employed should be calculated in accordance with the fiber used and the diameter of the vein to be treated; for a radial fiber, 7 J should be used for each millimeter of vessel diameter.^2^ If the fiber used is conventional, the recommended LEED is 10 J for each millimeter of vessel diameter.^2^ Therefore, LEED calculated based on saphenous diameter will result in high energy levels for large caliber saphenous veins. Adverse effects, such as pain, hematoma, and paresthesias, are energy-dependent and increase drastically at LEED > 100 J/cm.^2^^,^ 11

In order to achieve effective intravenous ablation results, sufficient energy should be applied to provoke denaturing of the collagen in the venous wall. This process takes place at temperatures from 70 to 100 ºC. Therefore, during the ablation process, it is necessary to administrate energy capable of generating elevated temperatures to effectively close the venous segment being treated.^12^^,^ 13

Some factors that may have influenced our high occlusion rate were: all patients were subjected to tumescence of the vein to be treated; all were in the Trendelenburg position; and a 600 micron radial fiber was used. The larger the diameter of the fiber, the higher the final temperature reached and the better the distribution and conduction of heat to the wall of the vein.^10^ The laser fiber catheter tip reaches 800-900 degrees, which falls to 90-100 degrees at a 4 mm distance, which is why the tip of the catheter must be kept in contact with the wall of the vein being treated.^2^ Additionally, presence of blood within the vein reduces the thermal ablation temperature and its efficacy. This is the reason for the importance of good tumescence and use of the Trendelenburg position, to empty the saphenous vein being treated and achieve high occlusion rates.

The venous occlusion rates in our sample of patients reduced over the course of follow-up. There are studies showing that advanced clinical stage and saphenous vein diameters exceeding 5 mm were strongly predictive of recanalization and greater propensity to needing a secondary procedure.^1^^,^ 5 ^,^ 11 Another study showed that males and those with reflux at the saphenofemoral junction also had higher rates of recanalization.^14^ Data on patients who had relapse were not analyzed separately in our study. It is possible that because of the chronic and progressive nature of the disease, rates of venous occlusion reduced over time because of the need for additional interventions in some patients.

There are studies showing that endolaser for saphenous veins smaller than 10 mm has a success rate of almost 100% and that when the same energy was used for patients with saphenous veins larger than 10 mm, the success rate fell to 70%.^1^^,^ 15 ^,^ 16 American Venous Forum guidelines recommend that thermal ablation with lasers is not appropriate for saphenous veins smaller than 2 mm or larger than 15 mm.^3^ However, these diameters are not described as absolute contraindications.^3^ There is still a need for studies to determine the ideal cutoff diameter for using endolaser.

Patients with advanced chronic venous disease (CEAP C3-C6) have saphenous veins of larger diameter, which is a reflection of the progressive nature of the disease.^11^ Moreover, patients with more serious varicosities are more prone to need a secondary procedure, irrespective of their CEAP class and regardless of the technique employed.^11^ The severity of varicose veins may not be correlated with the degree of venous disease, but it is indicative of which patients may need secondary procedures.^17^

Rates of recurrence after thermal ablation are similar to those after vein stripping, although the causes of recurrence are different. After vein stripping, the most common cause is neovascularization, whereas after thermal ablation it is recanalization of the vein treated and incompetence of the anterior accessory saphenous vein.^18^

This study is subject to limitations: first, associations between occlusion rates and diameters of saphenous veins were not analyzed because the data necessary had not been recorded. The study was limited to a single center, which reduces its external validity. Another limitation was considerable loss of patients over the course of follow-up. Losses primarily occurred from 3 years after surgery onwards. This could have influenced the results for long-term patency. This was also a study with retrospective analysis and without a control group.

The occlusion rate of saphenous veins treated with endolaser was lower in the presence of lipodermatosclerosis and in patients at advanced CEAP stages. Therefore, in these patients, it is probable that more energy should be applied to ensure that fibrotic transformation of the venous segment occurs and the treatment is effective. This correlation should be confirmed in comparative prospective studies.

This study informs the specialist that patients with advanced venous disease may exhibit lower rates of successful treatment of saphenous reflux with endolaser. In order to avoid unsuccessful treatment and/or early relapses, practitioners should be alert to these patients and take certain precautions. Apply more energy, induce tumescence, employ the Trendelenburg position, and utilize a radial fiber exceeding 600 microns are effective and safe measures.
