# Supplementary material for: Results of venous reflux treatment with 1,470 nm endolaser and correlation with degree of venous insufficiency
Source: J Vasc Bras. 2021 Apr 28;20:e20200172. doi: 10.1590/1677-5449.200172 (PMC8147881; doi:10.1590/1677-5449.200172)
Supplement: Translation

# Translation: Resultados do tratamento do refluxo de veias safenas com *endolaser* 1.470 nm e correlação com o grau de insuficiência venosa

**Authors:** Ana Paula Pires Silva, Daniel Mendes Pinto, Vanessa Aline Miranda Vieira Milagres, Leonardo Ghizoni Bez, Júlio César Arantes Maciel, Caetano de Souza Lopes

**Affiliations:** 1 Hospital Felício Rocho, equipe de Angiologia e Cirurgia Vascular, Belo Horizonte, MG, Brasil.; 2 Faculdade Ciências Médicas de Minas Gerais, Belo Horizonte, MG, Brasil.

**Keywords:** varizes, laserterapia, veia safena

## Abstract

**Contexto:**

Pacientes com doença venosa crônica avançada são mais propensos a exigir outros procedimentos para recidiva de veias varicosas. Ainda não está estabelecido se a gravidade da insuficiência venosa é um fator que influencia a taxa de oclusão de veias safenas tratadas por *endolaser*.

**Objetivos:**

Analisar a taxa de oclusão dos segmentos venosos tratados com *endolaser* e correlacionar com o *Venous Clinical Severity Score* (VCSS) e a classificação Clínica-Etiológica-Anatômica-Patológica (CEAP) dos pacientes.

**Métodos:**

Análise retrospectiva de coorte de pacientes operados com *endolaser* 1.470 nm entre novembro/2012 a março/2020. Foram realizadas estatística descritiva e curva de sobrevida de Kaplan-Meier com regressão de Cox para grupos de VCSS e CEAP.

**Resultados:**

Foram analisados 170 pacientes e 180 segmentos venosos; a idade média foi de 44,3 ± 9,2, sendo a maioria do sexo feminino (71%). A densidade de energia média utilizada na veia safena magna foi 49,2 ± 8,3 J/cm. As principais complicações foram dor no trajeto da safena (12,2%) e parestesias após 6 meses (17,2%). Não houve diferença na taxa de oclusão venosa entre grupos com VCSS ≤ 7 e VCSS > 7 (p = 0,067). O grupo de pacientes com CEAP agrupada C4-C5-C6 teve taxa de oclusão menor em relação ao grupo C2-C3 [*hazard ratio* (HR) = 3,22; intervalo de confiança (IC) 1,85, 5,61; p = 0,001].

**Conclusões:**

As taxas de oclusão de segmentos venosos tratados com *endolaser* foram menores na presença de classificações CEAP avançadas. Nesses pacientes, provavelmente deve-se despender mais energia para o tratamento eficaz das safenas.

## INTRODUÇÃO

As varizes dos membros inferiores são frequentemente secundárias à insuficiência das veias safenas. Entre 37 a 46% dos pacientes com doença venosa crônica possuem insuficiência do sistema venoso superficial^1^. A ablação térmica endovenosa a *laser* (*endovenous laser ablation*, EVLA) ou por radiofrequência são técnicas minimamente invasivas que reduzem algumas desvantagens associadas à cirurgia convencional, como hematomas, cicatrizes, neovascularização inguinal e tempo prolongado de recuperação no pós-operatório^1^. Além disso, é um procedimento menos invasivo que possibilita o tratamento no ambiente ambulatorial, utilizando apenas anestesia local^2^. A EVLA ou a ablação por radiofrequência são consideradas tratamentos de primeira linha para o refluxo de veias safenas^2^^,^ 3.

O diâmetro da veia safena não está relacionado com o grau de sintomatologia dos pacientes^1^, porém existem estudos que mostraram que as veias safenas magnas acima de 5,05 mm tiveram um valor preditivo positivo para incompetência da veia safena e podem apresentar uma fraca relação com o grau em que a doença se encontra^1^^,^ 4. Pacientes com doença venosa caracterizados pela classificação Clínica-Etiológica-Anatômica-Patológica (CEAP) C3 a C6 apresentaram 2 a 3 vezes mais chances de desenvolver recanalização no período de um ano^5^^,^ 6. Entretanto, não está definido se a doença venosa avançada pode interferir nos resultados da técnica de ablação endovenosa ou mesmo se existe maior risco de complicações desses pacientes.

O objetivo deste estudo foi analisar as taxas de oclusão das veias safenas com o *endolaser* de 1.470 nm e verificar se houve diferença destas conforme o grau da doença venosa pelas classificações *Venous Clinical Severity Score* (VCSS) e CEAP. Além disso, procuramos analisar se houve diferença nas taxas de complicações e nas taxas de recanalizações das veias safenas em 7 anos de seguimento dos pacientes. Definimos como hipótese a ser estudada a presença de correlação entre o grau de insuficiência venosa com a taxa de oclusão de safenas tratadas por EVLA.

## MÉTODO

Este estudo é uma coorte não concorrente de pacientes com varizes relacionadas à insuficiência de veias safenas tratadas por EVLA. Foi realizada uma análise retrospectiva dos pacientes com insuficiência venosa crônica tratados pela técnica EVLA entre novembro de 2012 a março de 2020. Os dados foram coletados pelo prontuário eletrônico do hospital e registro próprio da equipe de cirurgia vascular. O projeto foi aprovado pelo Comitê de Ética da instituição sob o parecer número 4.027.149.

Para o cálculo do tamanho amostral, consideramos uma análise não paramétrica da amostra, nível de significância de 0,05%, poder do teste estatístico de 80% e tamanho do efeito médio. Assim, para comparação entre os grupos, o menor número em cada grupo seria de 88, totalizando uma amostra de 176 no total.

Foram incluídos no estudo os pacientes submetidos a EVLA em veias safenas magna, parva, acessória e veia comunicante intersafena. Os critérios de exclusão foram pacientes com idade inferior a 18 anos e superior a 80 anos e pacientes com veias safenas superiores a 12 mm.

A técnica cirúrgica utilizada foi a EVLA, com o uso de *endolaser* de 1.470 nm com a fibra radial de 600 micra. A densidade de energia endovenosa linear alvo (*linear endovenous energy density*, LEED) foi de 40 a 60 J/cm. Os pacientes foram submetidos a flebectomia concomitante após a ablação da safena. Todos os pacientes foram submetidos a tumescência no trajeto da veia a ser tratada, feita com soro fisiológico associado ou não a xilocaína 2% sem vasoconstritor e, no momento, da termoablação estavam em posição de Trendelenburg. Os procedimentos foram realizados em bloco cirúrgico no período da manhã; os pacientes foram liberados após um período de observação variável entre 3 e 6 horas após o procedimento.

Os dados clínicos analisados foram sexo, idade, segmentos venosos acometidos, grau de insuficiência venosa pela classificação CEAP, grau de classificação pelo VCSS e presença de refluxo venoso. As variáveis do tipo de tratamento coletadas foram a quantidade de energia *laser* despendida, medida em J/cm, o segmento tratado e o tipo de anestesia utilizada. As variáveis de pós-operatório coletadas foram a presença de refluxo venoso ao Doppler-ultrassom do segmento venoso tratado, as complicações pós-operatórias precoces e tardias e os sintomas principais do paciente.

O acompanhamento foi realizado em consultório e em ambulatório pela equipe da pesquisa. A visita de seguimento foi programada para 7 dias no pós-operatório, 30 dias e a cada 6 meses. Em cada visita, era feita a avaliação clínica dos pacientes e a avaliação com ultrassom-Doppler dos membros inferiores. A recidiva de refluxo no segmento venoso tratado foi definida como a presença de refluxo acima 0,5 segundos às manobras de compressão muscular e com o paciente em ortostatismo. A recidiva de refluxo no nível da crossa da safena magna foi definida como a presença de refluxo além de 5 cm da junção safeno-femoral^7^.

Realizamos estatística descritiva dos dados, e o desfecho binário estudado foi a presença ou a ausência de refluxo venoso no segmento tratado com *endolaser*. Os dados dos pacientes foram divididos em seis grupos conforme a classificação clínica CEAP. Foram realizadas curvas de evento-desfecho de Kaplan-Meier para cada grupo da classificação CEAP. A diferença entre os grupos foi analisada pelo teste de log-rank e calculada a razão de risco (*hazard ratio*, HR) com intervalos de confiança de 95% para cada grupo por meio regressão multivariada de Cox. Os dados da gravidade da doença venosa medida pelo VCSS também foram divididos em grupos, e foi feita a regressão multivariada de Cox. O nível de significância para definir diferença estatisticamente significativa foi de 5%. Os *softwares* estatísticos utilizados foram os Graphpad Prism versão 8 (GraphPad Software, Califórnia, Estados Unidos) e Minitab versão 17 (Minitab, LLC, Pensilvânia, Estados Unidos).

## RESULTADOS

Entre novembro de 2012 a março de 2020, foram considerados 384 pacientes elegíveis. Os pacientes elegíveis foram os com insuficiência venosa crônica e indicação de intervenção cirúrgica. Desse total de pacientes, 170 foram submetidos ao tratamento com EVLA, totalizando 180 segmentos venosos tratados com EVLA. O número de pacientes incluídos, excluídos e o seu seguimento foram representados na Figura 1.

**Figura 1:** Fluxograma dos pacientes submetidos ao tratamento com ablação térmica endovenosa a *laser* (EVLA). IVC = insuficiência venosa crônica.

O número total de pacientes foi dividido entre 120 mulheres (71%) com média de idade de 53,68 ± 13,77 anos e 50 homens (29%) com média de idade de 50,10 ± 13,14 anos (p = 0,89). A anestesia utilizada em 102 casos foi a anestesia local com sedação anestésica (60%); em 40 casos, foi o bloqueio raquidiano (23,5%); e, em 28 casos, foi a anestesia geral (16,5%). A Tabela 1 mostra a distribuição dos segmentos venosos tratados e a energia utilizada. A safena magna foi o segmento venoso mais comumente tratado e a energia média dispendida foi de 49,12 ± 8,32 J/cm^2^.

**Tabela 1:** Distribuição dos 180 segmentos venosos tratados com *endolaser* 1.470 nm e energia utilizada.

Segmento venoso tratado	n (%)	Densidade de energia
		(LEED) (J/cm^2^)
Veia safena magna	161 (89)	49,12 ± 8,32
Veia safena parva	16 (9)	29,22 ± 5,11
Veia safena acessória	2 (1)	27,60 ± 10,55
Veia comunicante intersafena	1 (1)	25,11 ± 5,23

LEED: *linear endovenous energy density* (média ± desvio padrão).

A complicação mais comum foi a parestesia persistente 6 meses após o tratamento, que ocorreu em 17,22% dos casos, seguida da dor no trajeto da safena em 12,22%. Ocorreram três casos de trombose venosa profunda, todos diagnosticados entre 7 e 30 dias após a cirurgia (Tabela 2).

**Tabela 2:** Complicações após termoablação com *endolaser* 1.470 nm em 170 pacientes.

Complicação	n (%)
Parestesia após 6 meses	31 (17,22)
Dor no trajeto da safena magna	22 (12,22)
Pigmentação persistente após 6 meses	12 (6,67)
Trombose venosa profunda	3 (1,67)
Necrose de pele	0
Embolia pulmonar	0

A Tabela 3 mostra a taxa de oclusão dos segmentos venosos tratados durante o seguimento. Aos 30 dias, 175 dos 180 segmentos venosos tratados estavam ocluídos ao exame ultrassom-Doppler, configurando uma taxa de oclusão venosa de 97,22%. A taxa de oclusão dos segmentos venosos manteve-se acima de 95% até o segmento de 2 anos. Após 2 anos, nota-se uma redução do número de pacientes com os quais foi feito o seguimento, coincidente com a redução da taxa de oclusão para valores abaixo de 90%.

**Tabela 3:** Taxa de oclusão venosa durante o seguimento de 180 segmentos venosos tratados com *endolaser* 1.470 nm.

Tempo de seguimento	n	Segmentos ocluídos	% oclusão
30 dias	180	175	97,22%
6 meses	178	176	98,88%
1 ano	158	155	98,10%
2 anos	145	141	97,24%
3 anos	78	69	88,46%
4 anos	67	60	89,55%
5 anos	26	22	84,62%
6 anos	15	13	86,67%
7 anos	9	8	88,89%

A mediana do VCSS dos 170 pacientes foi 7. Os pacientes foram divididos em dois grupos: aqueles com VCSS ≤ 7 e aqueles com VCSS > 7. Foi feita a análise da taxa de oclusão dos segmentos venosos entre os dois grupos. A Figura 2 mostra a análise temporal utilizando a curva de evento-desfecho de Kaplan-Meier, comparando o resultado entre os dois grupos divididos pela mediana do VCSS. Não houve diferença na taxa de oclusão durante o seguimento de até 5 anos entre aqueles que apresentavam VCSS ≤ 7 e os que apresentavam VCSS mais elevado (p = 0,067).

**Figura 2:** Taxa de oclusão dos segmentos venosos entre grupos divididos pela mediana do *Venous Clinical Serverity Score* (VCSS).

A Figura 3 mostra o resultado da oclusão dos seguimentos venosos conforme o grau da classificação clínica CEAP. Quando analisados individualmente e durante o seguimento de até 5 anos, não houve diferença entre os graus de C2 a C6 quanto à taxa de oclusão venosa.

**Figura 3:** Taxa de oclusão dos segmentos venosos entre grupos divididos pela classificação Clínica-Etiológica-Anatômica-Patológica (CEAP).

Quando analisados conjuntamente, o grupo de pacientes classificados com C2 ou C3 apresentou taxa de oclusão dos segmentos venosos maior que o grupo de pacientes classificados com C4, C5 ou C6 (p < 0,001; HR = 3,22). Durante o tempo de seguimento de até 72 meses, a taxa de oclusão no grupo C2-C3 foi 3,22 vezes maior que o grupo de pacientes C4-C5-C6, resultado representado na Figura 4.

**Figura 4:** Taxa de oclusão dos segmentos venosos entre grupos selecionados através da classificação clínica. CEAP = Classificação Clínica-Etiológica-Anatômica-Patológica; HR: *hazard ratio*.

## DISCUSSÃO

Este estudo mostra que houve associação entre os graus avançados da classificação CEAP com as taxas menores de oclusão de veias safenas tratadas por EVLA. No entanto, a gravidade da insuficiência venosa medida pelo VCSS não foi um fator que influenciou, ao longo do tempo, o resultado da termoablação. Observamos também que as taxas de oclusão dos segmentos venosos reduziram durante o tempo de seguimento.

Nossos dados mostram que pacientes com dermatofibrose, úlceras cicatrizadas ou úlceras ativas em membros inferiores tiveram um risco de 3,22 vezes maior de recanalização que pacientes em classes iniciais de insuficiência venosa. Em um estudo, os autores demonstram que, em pacientes com insuficiência venosa, as camadas íntima e média das varizes são significativamente mais espessas e apresentam caráter progressivo conforme o avanço da doença venosa^5^. O espessamento da parede da veia pode resultar em dano transmural menos efetivo com subsequente recanalização da veia após EVLA^8^. Além disso, os pacientes com classe clínica C3 a C6 desenvolvem hipertensão venosa contínua e são mais propensos a remodelação da parede venosa. Essa hipertensão leva a alterações histológicas conhecidas como microangiopatia hipertensiva venosa, ativando a cascata inflamatória e se propagando para a microcirculação e os tecidos circundantes^9^. É possível que pacientes com doença venosa avançada apresentem alterações histológicas que impliquem em menor transformação fibrótica após a transferência de energia *laser* para a parede venosa.

O VCSS leva em conta os sintomas clínicos fornecidos pelos doentes, o que leva a informações variáveis para cada paciente, como, por exemplo, o grau de dor e o impacto da doença nas atividades de rotina. Estudos indicam que a sintomatologia dos pacientes e a classificação VCSS não apresentam relação direta proporcional com o grau em que a doença se encontra^1^^,^ 4. Essas variações podem ter influenciado nos resultados, justificando o nosso achado de VCSS avançados não interferirem nas recanalizações a longo prazo.

O tipo de anestesia mais utilizado nesta coorte de pacientes foi anestesia local com sedação, o que evidencia um procedimento menos invasivo, uma recuperação pós-operatória mais rápida e um procedimento viável de ser realizado em ambiente ambulatorial. Todos os procedimentos foram realizados em hospital, e os casos com anestesia geral foram feitos com máscara laríngea e sedação profunda. No registro *Vascular Quality Initiative*, mostra que, em tratamentos de veias varicosas nos Estados Unidos, 54,4% das cirurgias foram realizadas em consultórios com recursos semelhantes a instalações hospitalares e 37,1% foram feitas em ambiente hospitalar^10^.

A LEED se tornou a referência para os médicos realizarem os cálculos de entrega de energia. O recomendado na literatura varia entre 40 e 90 J/cm de energia linear para a veia safena magna^1^. A LEED média utilizada nos nossos pacientes, 49,12 ± 8,32 J/cm, foi condizente com dados de outros autores. Velocidade de fluxo, histologia da parede venosa, calibre da veia, quantidade de veias tributárias, potência e tipo de fibra utilizada podem influenciar a quantidade de energia necessária^1^.

A energia a ser utilizada deve ser calculada de acordo com a fibra utilizada e o diâmetro da veia a ser tratada; para a fibra radial, deve-se utilizar 7 J para cada diâmetro do vaso em milímetro^2^. Se a fibra utilizada for a convencional, a LEED recomendada é 10 J por diâmetro do vaso em milímetro^2^. Assim, a LEED baseada no diâmetro da safena resultará em altas energias para safenas de grande calibre. Os efeitos adversos, como dor, hematomas e parestesias, são dependentes da energia e aumentam drasticamente com LEED > 100 J/cm^2^^,^ 11.

Para obter um resultado eficaz da ablação endovenosa, deve-se despender uma energia que provoque a desnaturação do colágeno da parede venosa. Esse processo ocorre em temperaturas entre 70 e 100 ºC. Portanto, no processo de ablação, é necessário administrar uma energia capaz de gerar temperaturas elevadas para o fechamento do segmento venoso tratado^12^^,^ 13.

Alguns fatores que podem ter influenciado nossa alta taxa de oclusão foram todos os pacientes terem sido submetidos a tumescência da veia a ser tratada e estarem em posição de Trendelenburg, além da utilização da fibra radial de 600 micras. Quanto maior o diâmetro da fibra, maior a temperatura final atingida e melhor é para a distribuição e condução de calor até a parede da veia^10^. A ponta do cateter da fibra do *laser* chega a 800-900 graus e, a 4 mm de distância, cai para 90-100 graus. Por esse motivo, a ponta do cateter tem que estar em contato com a parede da veia a ser tratada^2^. Além disso, a presença de sangue no interior da veia diminui a temperatura da ablação térmica e a eficiência. Por isso, a importância de uma boa tumescência e a posição de Trendelenburg para o esvaziamento da veia safena a ser tratada e, assim, para obter altas taxas de oclusão.

Em nossos pacientes, as taxas de oclusão venosas reduziram durante o tempo de seguimento. Há estudos que mostram que a classe clínica avançada e o diâmetro da veia safena maior que 5 mm foram fortes preditores de recanalização, mais propensos a necessitar de um procedimento secundário^1^^,^ 5 ^,^ 11. Outro estudo mostra que o sexo masculino e a presença de refluxo na junção safeno-femoral também apresentaram maiores taxas de recanalização^14^. Os dados dos pacientes que apresentaram recidiva não foram analisados de forma separada em nosso estudo. É possível que devido à cronicidade e ao caráter evolutivo da doença, as taxas de oclusão venosa tenham reduzido com o tempo devido à necessidade de novas intervenções em alguns pacientes.

Existem trabalhos que mostram que o *endolaser* para safenas menores que 10 mm apresentam taxa de sucesso quase 100%, e, quando utilizada a mesma energia para pacientes com safenas maiores que 10 mm, a taxa de sucesso caiu para 70%^1^^,^ 15 ^,^ 16. Orientações do *American Venous Forum* indicam que, em safenas menores que 2 mm e maiores que 15 mm, a termoablação com *laser* é inapropriada^3^. Entretanto, tais diâmetros não são descritos como uma contraindicação absoluta^3^. Ainda são necessários estudos indicando qual seria o limite de diâmetro ideal para o uso do *endolaser*.

Pacientes com doença venosa crônica avançada (CEAP C3-C6) apresentam veias safenas de maior diâmetro, refletindo, assim, a natureza progressiva da doença^11^. Além disso, pacientes com varicosidades mais graves, independentemente da classe CEAP, são mais propensos a exigir um procedimento secundário, qualquer seja a técnica a ser utilizada^11^. A gravidade das varizes pode não se correlacionar com o grau de doença venosa, mas é uma indicação de quais pacientes podem necessitar de procedimentos secundários^17^.

As taxas de recorrência da ablação térmica, quando comparada com a fleboextração, são semelhantes, sendo diferenciadas pela causa das taxas de recorrência. Na fleboextração, a causa mais comum é a neovascularização, e, na ablação térmica, é a recanalização da veia tratada e a incompetência da veia safena acessória anterior^18^.

Este estudo apresenta limitações: primeiro, não foram analisadas as taxas de oclusão associadas aos diâmetros das veias safenas devido à insuficiência de dados obtidos nos registros. O estudo foi limitado a somente um centro, o que reduz a sua validade externa. Outra limitação foi uma perda de seguimento considerável dos pacientes. A perda ocorreu principalmente após 3 anos da cirurgia. Isso pode ter influenciado nos resultados de perviedade a longo prazo. Além disso, foi um estudo com análise retrospectiva e sem um grupo controle.

A taxa de oclusão de safenas tratadas com *endolaser* foi menor na presença de dermatofibrose e em classificações CEAP avançadas. Dessa forma, nesses pacientes provavelmente deve-se despender mais energia para que ocorra a transformação fibrótica do segmento venoso e o tratamento seja eficaz. Tal correlação deve ser comprovada com estudos prospectivos comparativos.

O estudo informa ao especialista que pacientes com doença venosa avançada podem apresentar uma menor taxa de sucesso no refluxo de safenas com o *endolaser*. Para que não ocorra insucesso do tratamento e/ou recidivas precoces, deve-se estar atento a esses pacientes e tomar alguns cuidados. Despender mais energia, realizar tumescência, empregar a posição de Trendelenburg e utilizar a fibra radial acima de 600 micras são medidas eficazes e seguras.
